# Supplementary material for: When band convergence is not beneficial for thermoelectrics
Source: Nat Commun. 2021 Jun 8;12:3425. doi: 10.1038/s41467-021-23839-w (PMC8187731; doi:10.1038/s41467-021-23839-w)
Supplement: Supplementary file 1 — Supplementary Information [file 41467_2021_23839_MOESM1_ESM.pdf]

Supplementary Information: When Band Convergence is Not Beneficial for Thermoelectrics

Junsoo Park,[1,a] Maxwell Dylla,[2] Yi Xia,[2] Max Wood,[2] G. Jeffrey Snyder,[2,b] and Anubhav Jain[1,c]

[1] *Energy Technologies Area, Lawrence Berkeley National Laboratory, Berkeley, CA 94720, USA*

[2] *Department of Materials Science & Engineering, Northwestern University, Evanston, IL 60208, USA*

(Dated: 18 May 2021)

I. SUPPLEMENTARY FIGURES

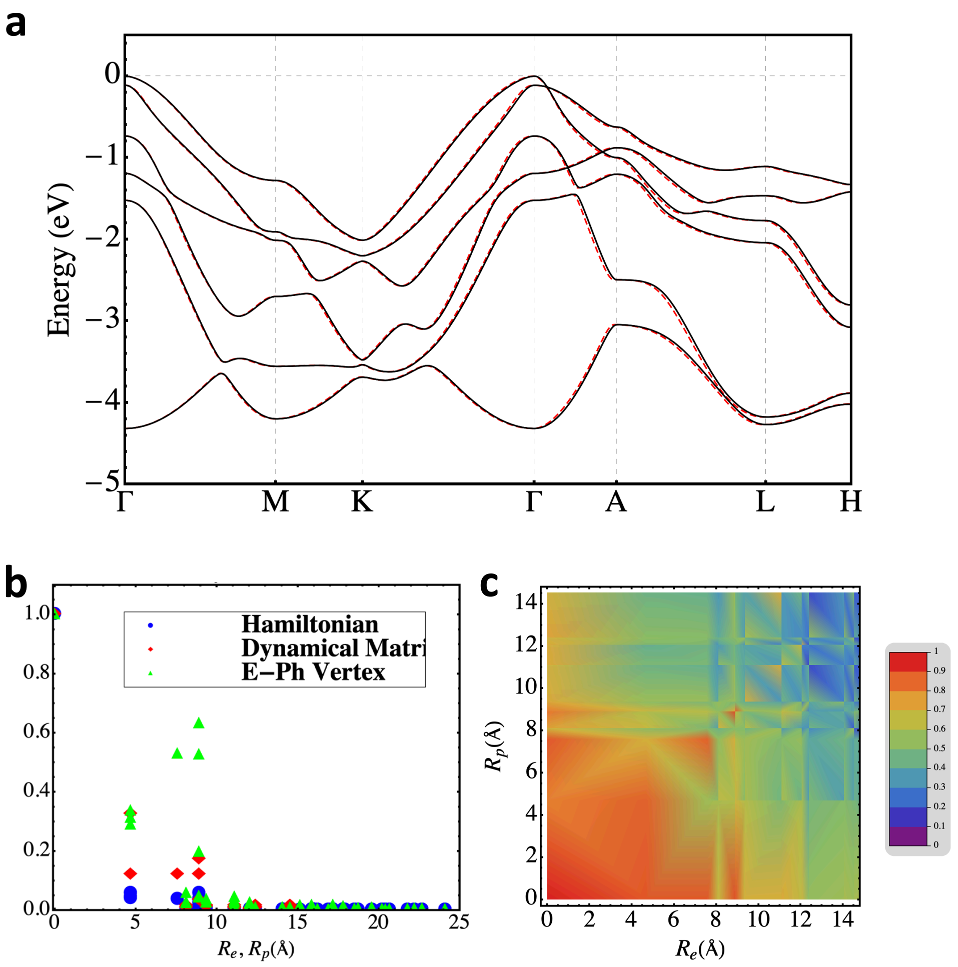

FIG. S1. **Wannier interpolation quality check. a)** Wannier-interpolated valence band structure (dotted red) excellently reconstructs DFT-calculation (black). **b)** Decay of interpolated quantities in the Wannier representation: decay of Hamiltonian and the e-ph matrix elements are with respect to electronic distance (R_e), and decay of the dynamical matrix is with phonon distance (R_p). **c)** Decay of the e-ph matrix elements in electronic and phonon distances.

[a] Electronic mail: qkwnstn@gmail.com

[b] Electronic mail: jeff.snyder@northwestern.edu

[c] Electronic mail: ajain@lbl.gov

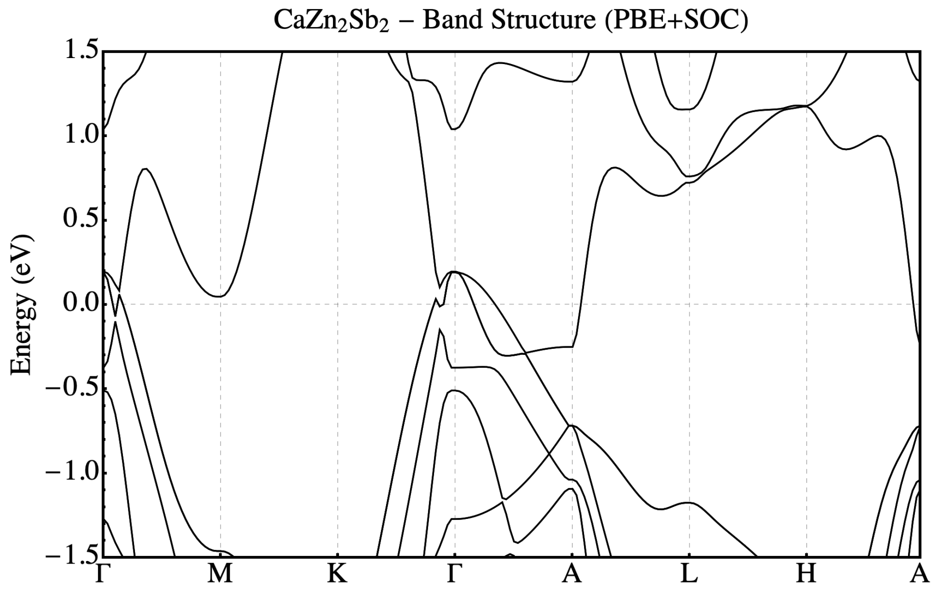

FIG. S2. **The PBE+SOC band structure of $CaZn_2Sb_2$**. It is incorrectly metallic and not amenable to EPW study.

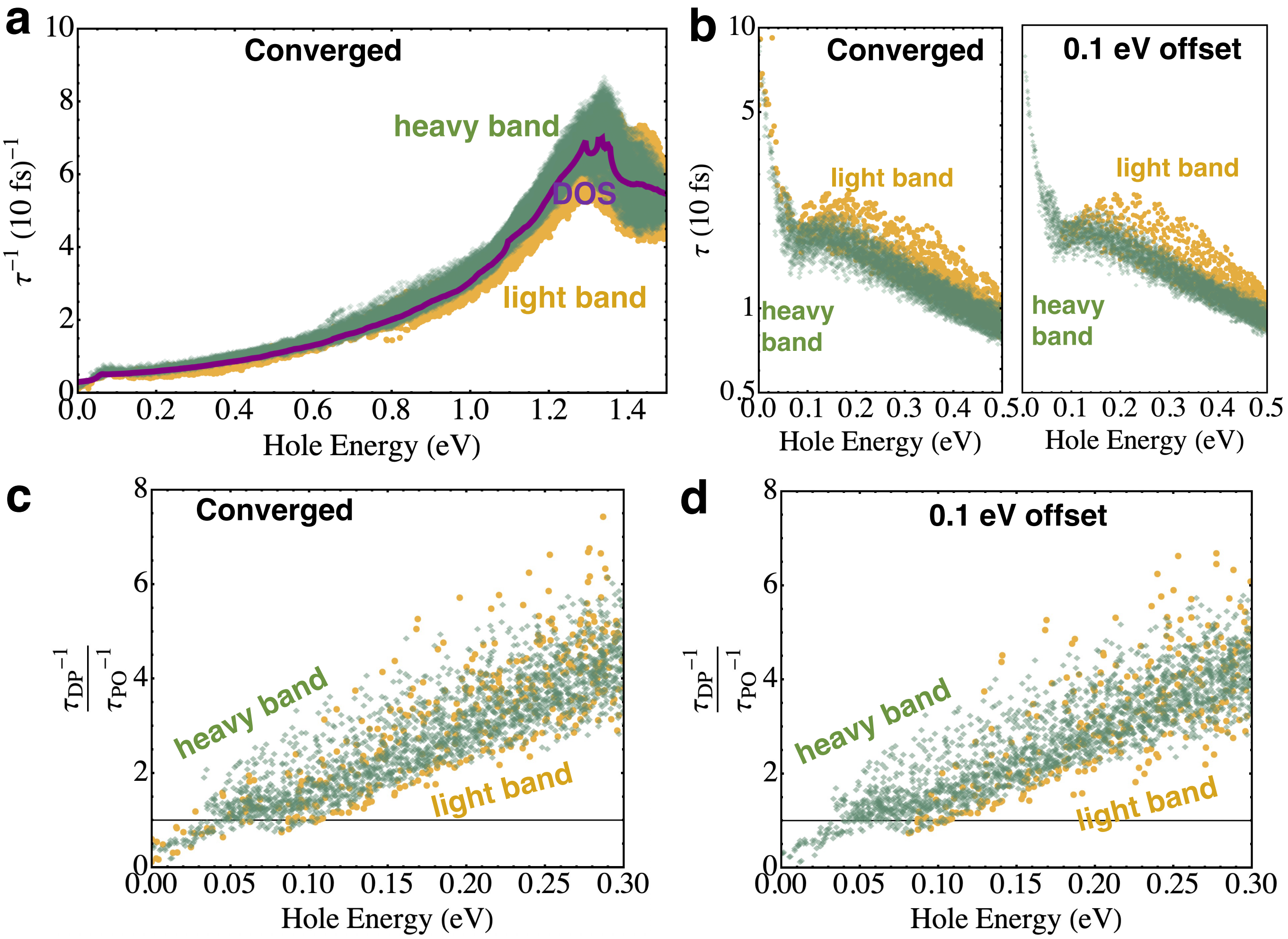

FIG. S3. **Additional information on scattering in $CaZnMgSb_2$ at 600 K. a)** The overall scattering rates, where DOS is overlaid in purple. **b)** The overall hole lifetimes with band convergence and with 0.1 eV offset. **c)** The ratio of DPS to POS after convergence and **d)** before convergence with 0.1 eV offset.

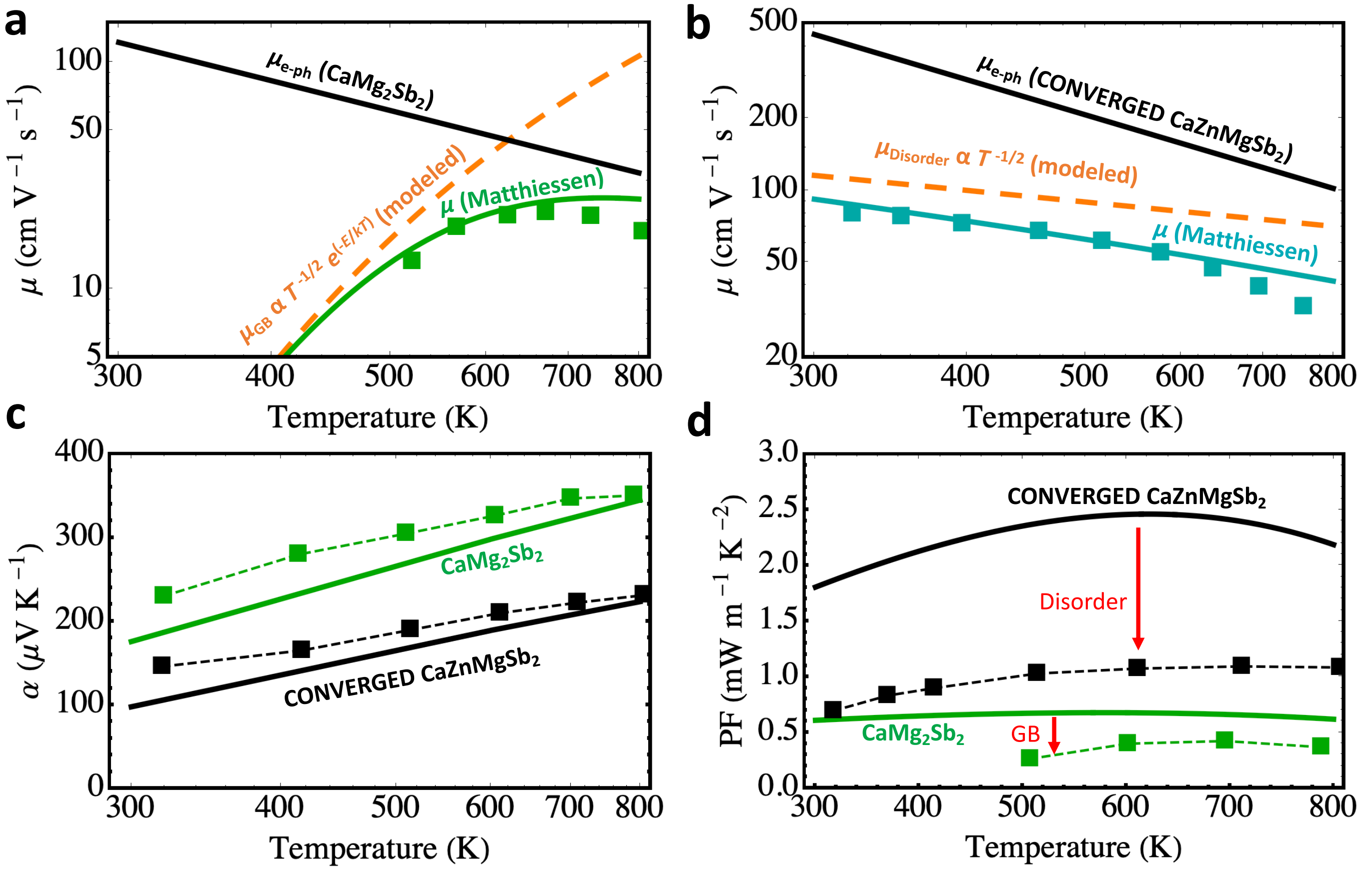

FIG. S4. **Additional information on transport of $CaZnMgSb_2$ at 600 K. a–b)** Computed e-ph-limited mobilities, modeled grain-boundary-limited and disorder-limited mobilities, and the overall theoretical mobilities approximated by Matthiessen's rule, plotted against experimental data points at their respective experimental carrier concentration. **a)** $CaMg_2Sb_2$ at $n_h = 10^{19}$ cm^{-3}. **b)** $CaZnMgSb_2$ at $n_h = 3 \times 10^{19}$ cm^{-3}. **c)** Computed e-ph-limited Seebeck coefficients of $CaMg_2Sb_2$ and band-converged $CaZnMgSb_2$ plotted against experimental data points (squares). **d)** Computed e-ph-limited power factors of $CaMg_2Sb_2$ and band-converged $CaZnMgSb_2$ plotted against experimental data points (squares).

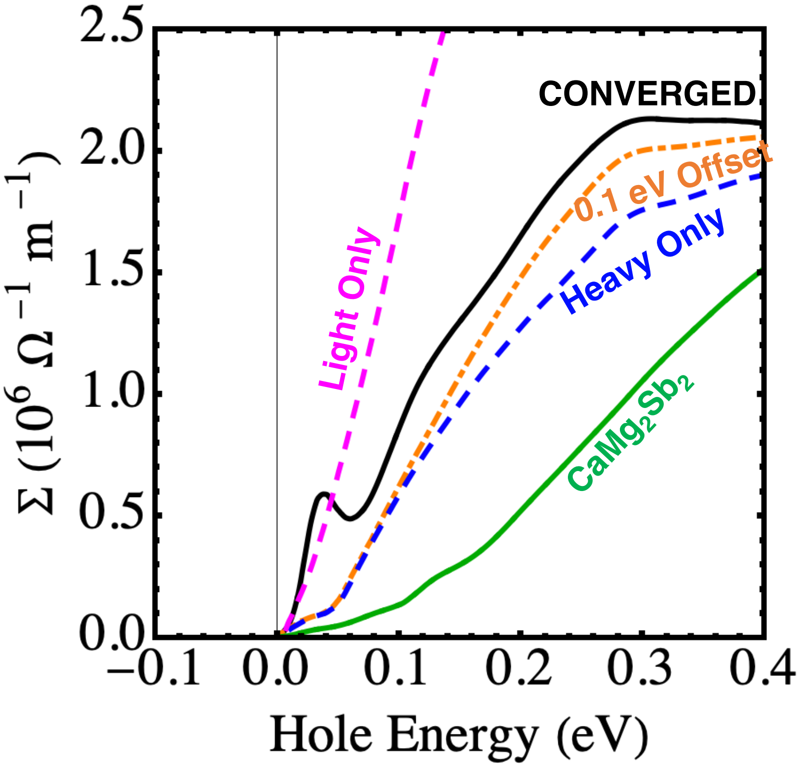

FIG. S5. **Spectral conductivity $\Sigma(E) = v^2(E)\tau(E)D(E)$ of the band configurations at 600 K.**

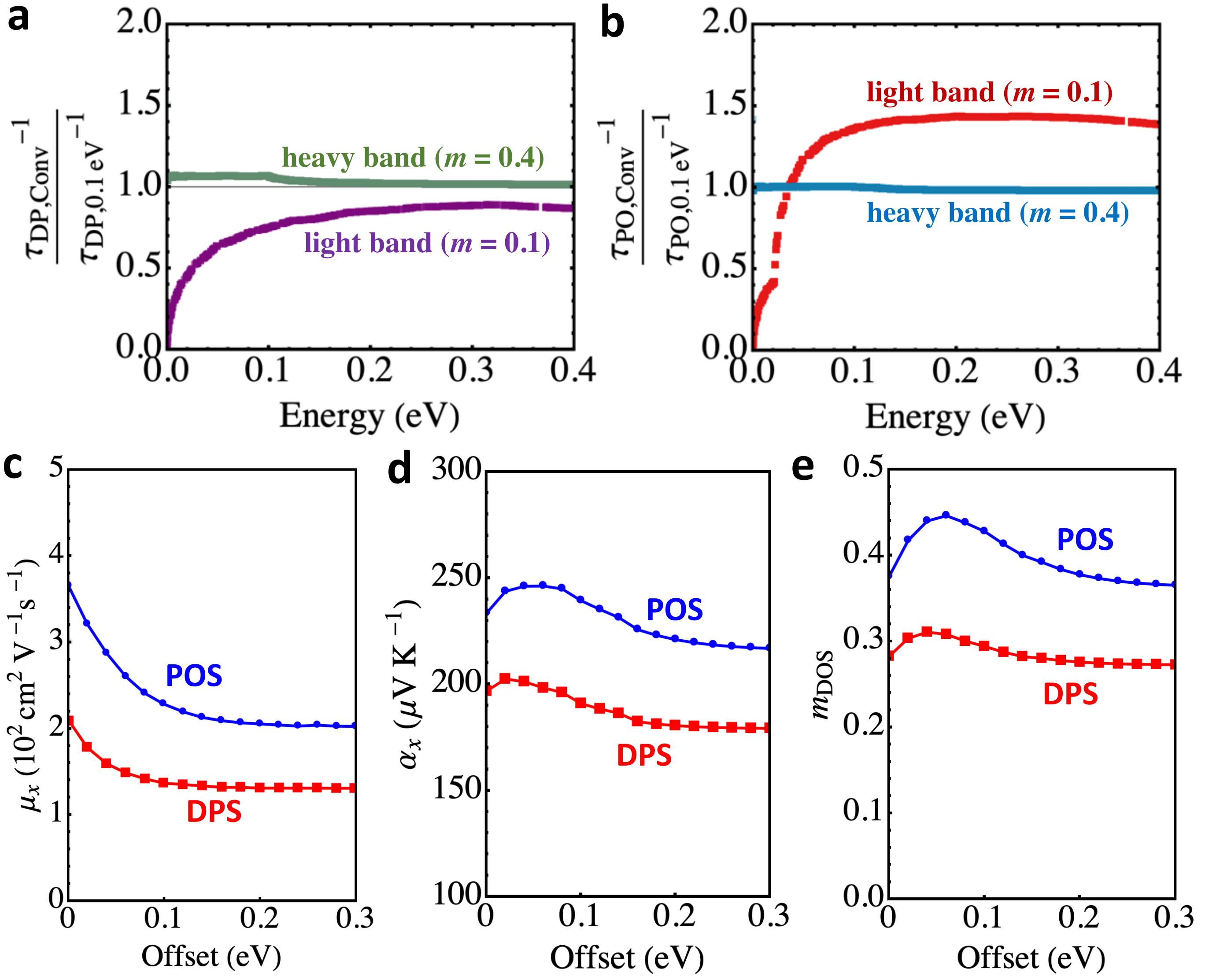

FIG. S6. **Model scattering and transport properties at 600 K.** The model band structures and the scattering model are from Ref. 1. The band curvature effective masses of the upper and lower bands are 0.4 and 0.1 respectively, which are nearly equivalent to the corresponding values in $CaZnMgSb_2$ alloy. At each offset, the carrier concentration is optimized. **a)** The ratio of DPS and **b)** the ratio of POS before and after convergence by closing a 0.1 eV offset. **c)** Mobility increases with band convergence because the lighter band rises. **d)** The Seebeck coefficient maximizes short of full convergence. **e)** The DOS effective mass closely follows the Seebeck profile.

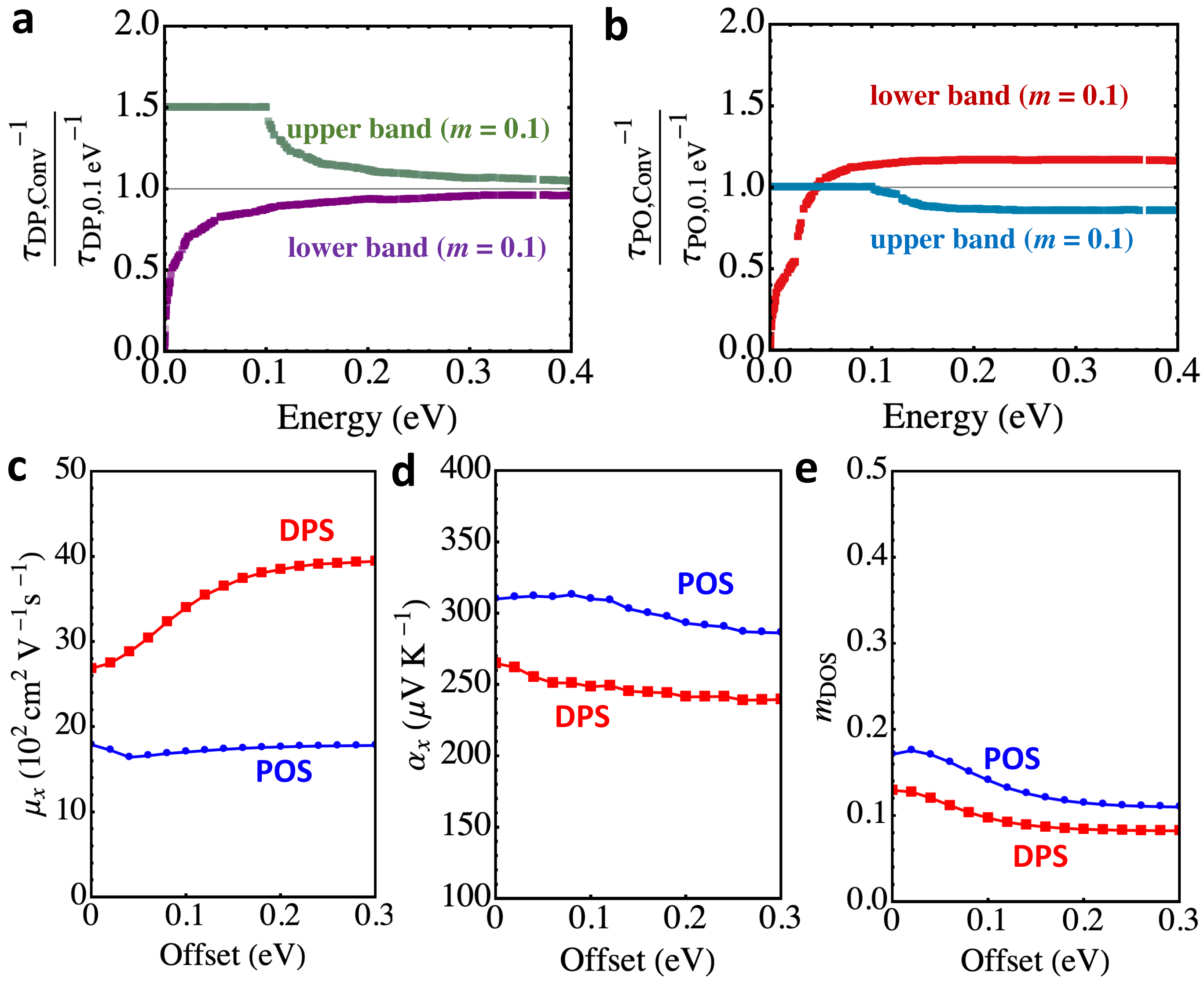

FIG. S7. **Additional model scattering and transport properties at 600 K based on Ref. 1.** The two bands of identical curvature effective masses of 0.15. respectively. At each offset, the carrier concentration is optimized. **a)** The ratio of DPS and **b)** the ratio of POS before and after convergence by closing a 0.1 eV offset. **c)** Mobility decreases with band convergence in the DPS case because additional band means identical group velocity but heavier scattering due to interband transitions. **d)** The Seebeck coefficient essentially maximizes at full convergence. **e)** The DOS effective mass closely follows the Seebeck profile.

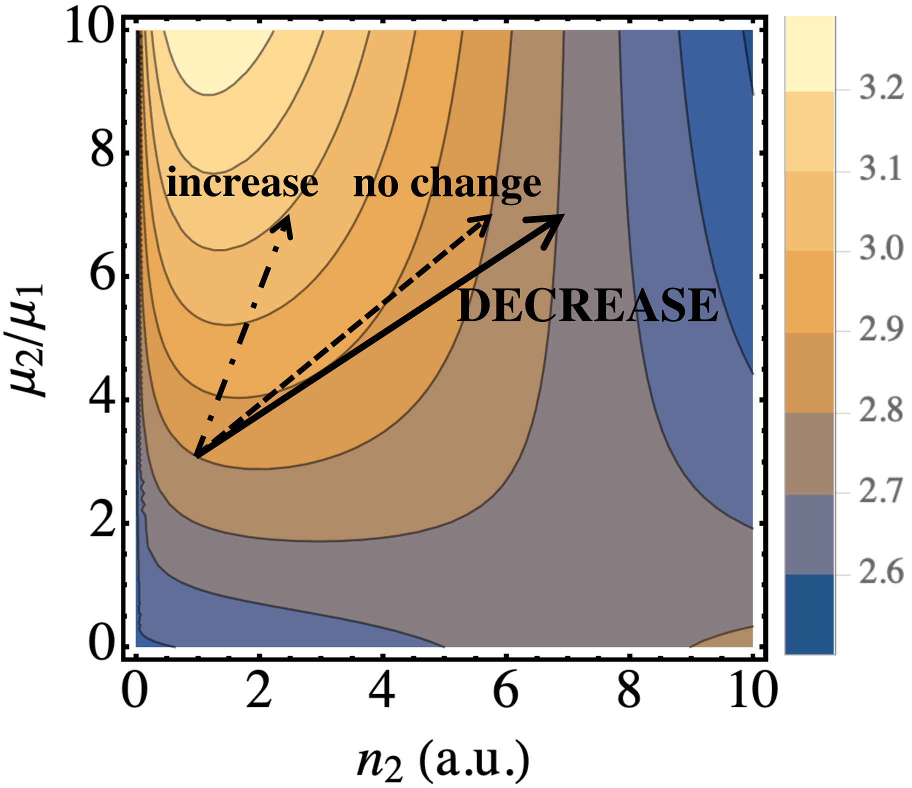

FIG. S8. **Contour plot of the two-band Seebeck coefficient (Eq. S7) as a function of n_2 and mobility ratio μ_2/μ_1.** We choose parameters that closely represent the Zintl alloy case. Band curvature effective masses are chosen to be $m_1 = 0.4$ and $m_2 = 0.1$. Since lower band moves up by 0.1 eV, n_2 would increase approximately by a factor of 7 during convergence at 600 K. This path is traced by the solid arrow. Fixing $n = 77$ obeys the parabolic band relation $n_1/n_2 = (m_1/m_2)^{3/2} = 10$ at the point of convergence where $n_1 = 70$ and $n_2 = 7$. The dashed and dot-dashed arrows indicate other cases in which the Seebeck coefficient can increase or stay the same during band convergence.

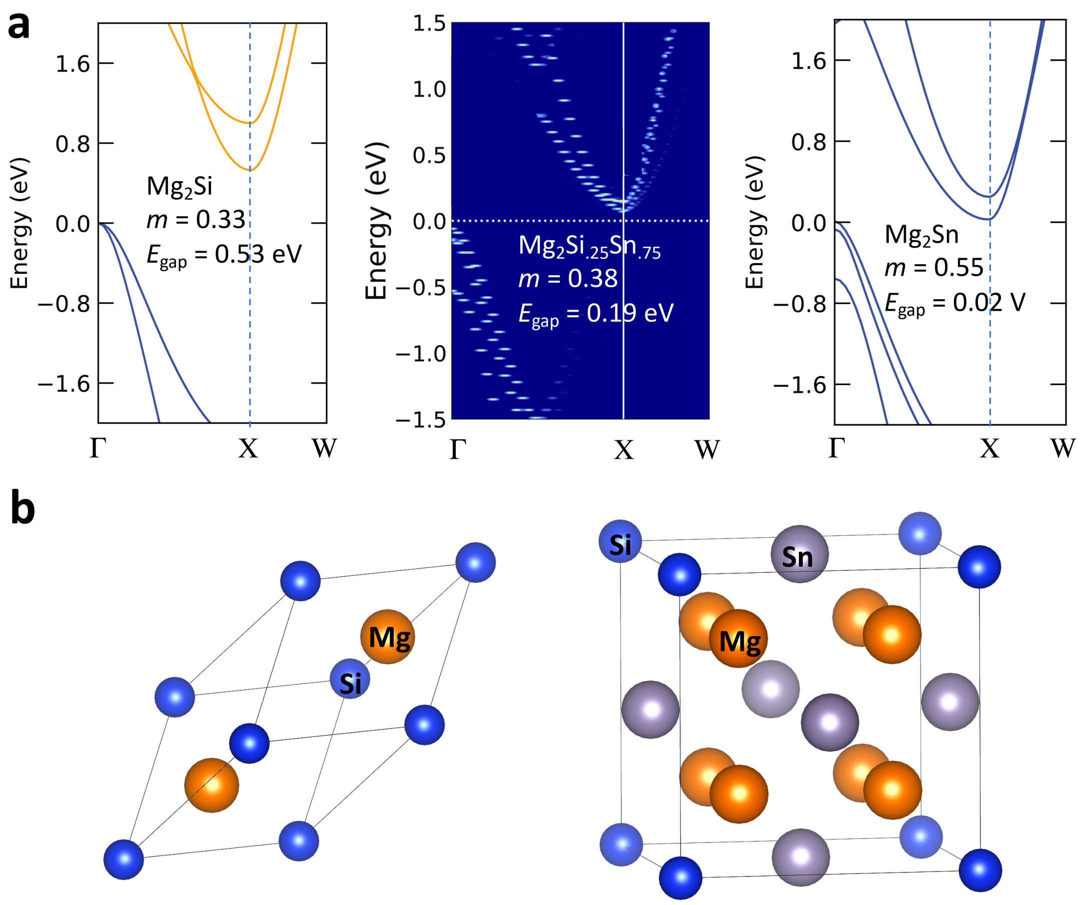

FIG. S9. **The $Mg_2Si_{1-x}Sn_x$ system.** The alloys perform yield higher PF than either end compound and undergo band convergence at one **k**-point at around $x \sim 0.7$. **a)** Band structures of the end compounds and $Mg_2Si_{0.25}Sn_{0.75}$. The alloy band structure is calculated using BandUP[2,3]. Numbers in the figure are the harmonic mean of directional curvature effective masses and the band gap. **b)** The primitive crystal structures of Mg_2Si and $Mg_2Si_{0.25}Sn_{0.75}$. The latter is simple cubic whereas the former is face-centered cubic.

II. SUPPLEMENTARY METHODS

All formulae are provided in Hartree atomic units: $\hbar = m_e = e = 4\pi\epsilon_0 = 1$

The matrix elements for scattering due to phonon deformation, the so-called "deformation-potential" scattering, are computed by DFPT as follows:

$$g^{\mathrm{DPS}}_{\nu'\nu\lambda\mathbf{kq}} = \left\langle \psi_{\nu'\mathbf{k}+\mathbf{q}} \left| \sum_{ajl} \sqrt{\frac{1}{2M_a\omega_{\lambda\mathbf{q}}}} e^{i\mathbf{q}\cdot\mathbf{R}_l} \frac{\partial V}{\partial u^{al}_{\mathbf{q}j}} \hat{\mathbf{u}}^{al}_{\lambda\mathbf{q}j} \right| \psi_{\nu\mathbf{k}} \right\rangle \tag{S1}$$

where ψ_ν and $\psi_{\nu'}$ are the initial and final electronic bands, ∂V is the perturbation in the potential due to phonon deformation, $\hat{\mathbf{u}}_{\lambda\mathbf{q}j}$ is phonon eigenvector for atom a, mode λ and Cartesian direction j, and u is atomic displacement, and M_a is the mass of atom a. The polar-optical scattering matrix elements are computed as follows:

$$g^{\mathrm{POS}}_{\nu'\nu\lambda\mathbf{kq}} = i\sum_a \sqrt{\frac{1}{2M_a\omega_{\lambda\mathbf{q}}}} \sum_{\mathbf{G}\neq-\mathbf{q}} \frac{(\mathbf{q}+\mathbf{G})\cdot\mathbf{Z}^*\cdot\hat{\mathbf{u}}_{a\lambda\mathbf{q}}}{(\mathbf{q}+\mathbf{G})\cdot\epsilon^\infty\cdot(\mathbf{q}+\mathbf{G})} \left\langle \psi_{\nu'\mathbf{k}+\mathbf{q}} \right| e^{i(\mathbf{q}+\mathbf{G})\cdot(\mathbf{r}-l)} \left| \psi_{\nu\mathbf{k}} \right\rangle, \tag{S2}$$

where $\mathbf{Z}^*$ is the Born effective charge tensor, ϵ^∞ is the high-frequency dielectric permittivity tensor, $\mathbf{G}$ is the reciprocal lattice vector, and $\mathbf{r}$ is atomic position vector. The total matrix elements are sums of the two: $g = g^{\mathrm{DPS}} + g^{\mathrm{POS}}$. Fig. S1 validates accurate Wanniner interpolation and proper localization of the interpolated quantities (band structure, phonon dynamical matrix, and the scattering matrix).

As the two original Mg sites are symmetry-equivalent, it matters not which one is occupied by Zn for $CaZnMgSb_2$. We omit calculation of the $CaZn_2Sb_2$ because it requires an advanced functional to capture its semiconducting character and therefore is not amenable to EPW. See Fig. S2 for its metallic PBE+SOC band structure.

III. SUPPLEMENTARY DISCUSSIONS

A. On the Seebeck Coefficient and Density-of-States Effective Mass

It may seem unintuitive at first that the DOS effective mass (m_{D}) decreases with band convergence, as convergence of bands increases the total DOS. We emphasize here that m_{D} is not uniquely determined by DOS or band profile, and is certainly not guaranteed to monotonically increase with the total DOS. Even if two bands belonging to two different systems had identical DOS and band curvature, their m_{D} could still be different if the dominant scattering mechanism were different between the two systems. Because $m_{\mathrm{D}} = \left(\frac{\mu_w}{\mu}\right)^{2/3}$, and weighted mobility (μ_w) is defined through the Seebeck coefficient (α) as per Eqs. 1–2 in the main text, it is the relative behaviors of α and μ that ultimately determine m_{D}. If scattering changes in behavior such that μ decreases and α increases, then m_{D} increases. If μ increases but α decreases, then m_{D} decreases.

It is a well known consequence that the Seebeck coefficient of a given band changes with the scattering mechanism. In the single parabolic band model[4],

$$\alpha = k_{\mathrm{B}}\left(\frac{E_{\mathrm{F}}}{k_{\mathrm{B}}T} - r - \frac{5}{2}\right) \tag{S3}$$

where r is the power of energy to which scattering rates scale. For instance, if $\tau^{-1} \propto E^{0.5}$ under one scattering mechanism whereas $\tau^{-1} \propto E^{-0.5}$ under another, α is higher under the latter scattering regime. Even if the overall lumped scattering rate were equal between the two processes such that they generate the same μ, because the energy-dependence of their rates remain different, α will be higher under the latter. Therefore, μ_w and m_{D} are both higher under the latter.

Now, let us first consider the typical manner in which band-convergence is conceived, where interband scattering is minimal or inexistent. In this case, additional pockets generally increase α at a given carrier concentration, and μ is nominally not altered due to band convergence. It follows then that increase in total DOS indeed leads to increase in m_{D}, as expected.

When opportunity for interband scattering is present, then scattering rates can no longer generally be modeled with a simple power of energy. It nevertheless remains true that certain scattering behaviors would be more beneficial for α than others. Interband scattering can change in behavior in a way α could still increase but also decrease,

which would be mirrored by μ_w and $m_{\rm D}$. In our Zintl case, as the light band arises to converge the heavy band at the valence band maximum, it increases μ (higher overall group velocity) but incurs changes in DPS and particularly POS that reduces α. Combination of higher μ but lower α and hence stagnant μ_w leads to decreased $m_{\rm D}$, in spite of increased total DOS. In short, whether the total DOS increases or decreases is irrelevant for $m_{\rm D}$, at least not directly, and affects $m_{\rm D}$ only through what it means for interband scattering.

To graphically portray the impact interband scattering has on α, we provide in Fig. S5 comparative spectral conductivitity of the Zintl band configurations. The profile of $\Sigma(E) = v^2(E)\tau(E)D(E)$ is what determines α, as dictated by Boltzmann transport formalism

$$\alpha = \frac{1}{T}\frac{\int \Sigma(E)(E_{\rm F}-E)\left(-\frac{\partial f}{\partial E}\right)dE}{\int \Sigma(E)\left(-\frac{\partial f}{\partial E}\right)dE}, \tag{S4}$$

where one may identify the denominator with Ohmic charge conductivity (σ). The only difference between the numerator and the denominator is the term $E_{\rm F}-E$ in the former, which stipulates that for α to be high, $\Sigma(E)$ should be relatively higher further away from the Fermi level (i.e. higher energies) to increase the numerator, and relatively lower near the Fermi level (i.e. lower energies) to decrease the denominator. Of note, this is the underlying reason why a smaller value of r is beneficial in Eq. S3 for single parabolic band, i.e., it is more beneficial for lifetimes to be higher (scattering to be weaker) at higher energies in relative terms. Also for these reasons has the rule-of-thumb that steeply increasing $\Sigma(E)$ benefits α has come to be. This, however, presumes that scattering rates (hence lifetiems) are simply behaved such that $\Sigma(E)$ increases monotonically with E. This presumption is not generally valid in the presence of interband scattering, as evidenced by Fig. S5

We in fact find that $\Sigma(E)$ of the band-converged configuration does not behave trivially. It starts out with an even steeper onset than that of the lower-band-only case owing to coexistence of the two bands supplying higher carrier concentration. However, it reverses course and dips around 0.07 eV corresponding to the portion of the light band that increases in overall scattering due to convergence, which is slightly past the emission onset for POS. Emission onset is the energy below which inelastic phonon emission is very weak if not outright forbidden due to absence of final states (e.g. electron at 30 meV cannot emit a phonon with a frequency of 50 meV because -20 meV is in the band gap). Thus, emission onset is usually close in value to the highest optical frequency. Thereafter, POS is fully activated, and $\Sigma(E)$ again increases with E but this time with a more gradual slope - similar to those of the 0.1-eV-offset and heavy-band-only cases, indicating that the heavy band dominates transport from there on. From the perspective of Eq. S4, this $\Sigma(E)$ profile of the band-converged case is not the most favorable for α. The protrusion at $E < 0.07$ eV increases the denominator of Eq. S4, improving σ and hence μ over the other band configurations. However, the dip that follows reduces the numerator of Eq. S4 relative to the denominator, reducing α.

We further support our observation by means of complementary modeling studies. Fig. S6 is based on Ref. 1, whereby realistic model band structures and modifications of analytic scattering models have been used. We use two bands with $m = 0.4$ and $m = 0.1$, whether the former is fixed atop and the latter shifts for convergence, for the semblance with the $CaZnMgSb_2$ bands in question. In Figs. S6a–b, the scattering rates are computed at 600 K for bands. Under DPS, the lower band only decreases and the upper band only increases in scattering due to convergence. Under POS, the lower band increases in scattering due to convergence past the emission onset. The results establish that the scattering behavior of DPS and POS during convergence computed for $CaZnMgSb_2$, in Fig. 3 of the main text, are generalizable. Figs. S6c–e qualitatively reproduce that α and $m_{\rm D}$ do not peak at full convergence but rather around 0.1 eV, even though μ continues to improve up to full convergence. Because α is different under the two scattering regimes, $m_{\rm D}$ is also different under the two regimes (through μ_w). That is, $m_{\rm D}$ is not uniquely determined by the band structure or DOS alone.

If as in Fig. S7 two identical bands are used instead, each with $m = 0.15$ and $m = 0.15$, then the behaviors are somewhat different. In this case, even with interband scattering. α continues to increase with convergence until full convergence as well as $m_{\rm D}$. These behaviors are consistent with the way in which band convergence is typically conceived. The underlying reason is that when the two bands share the same shape, then interband scattering cleanly increases by a constant factor upon full convergence, as opposed to when two bands have disparate curvature profiles. Nevertheless, μ decreases because the bands' identical shapes do not change group velocity while inviting more scattering. Of course, α and $m_{\rm D}$ still depend on the scattering regime.

In short summary, when two bands are identical, decrease in μ is the main limiting agent of the benefit of band convergence since α is expected to improve. On the other hand when a lighter band converges upon a heavier band, then decrease in α is the limiting agent while μ is expected to improve. This is the case for the Zintl alloy. If a heavier band converges upon a lighter band, the band convergence is likely to be harmful since the overall group velocity drops in addition to increased scattering.

Even more qualitative analysis can also establish the possible reduction of α due to band convergence. Given two

bands, the Seebeck coefficient can be expressed as

$$\alpha = \frac{\sigma_1\alpha_1 + \sigma_2\alpha_2}{\sigma_1 + \sigma_2} = \frac{n_1\mu_1\alpha_1 + n_2\mu_2\alpha_2}{n_1\mu_1 + n_2\mu_2}, \tag{S5}$$

where n denotes carrier concentration and subscript 1 and 2 respectively indicate the upper band (fixed) and the lower band (converging). Using the Seebeck coefficient expression for a degenerately doped single parabolic band,

$$\alpha = \frac{2k_{\mathrm{B}}^2 T}{3} m \left(\frac{\pi}{3n}\right)^{\frac{2}{3}}, \tag{S6}$$

we can recast Eq. S5 as follows:

$$\begin{aligned}\alpha &= \frac{2k_{\mathrm{B}}^2 T}{3}\left(\frac{\pi}{3}\right)^{\frac{2}{3}} \frac{m_1 n_1^{\frac{1}{3}}\mu_1 + m_2 n_2^{\frac{1}{3}}\mu_2}{n_1\mu_1 + n_2\mu_2} \\ &= \frac{2k_{\mathrm{B}}^2 T}{3}\left(\frac{\pi}{3}\right)^{\frac{2}{3}} \frac{m_1(n-n_2)^{\frac{1}{3}} + m_2 n_2^{\frac{1}{3}}\left(\frac{\mu_2}{\mu_1}\right)}{(n-n_2) + n_2\left(\frac{\mu_2}{\mu_1}\right)}.\end{aligned} \tag{S7}$$

Then for given m_1, m_2, and fixed total n (n=$n_1 + n_2$), the Seebeck coefficient can be modeled after Eq. S7 by using n_2 and μ_2/μ_1 as two tunable parameters. The result is illustrated in Fig. S8. As the lower band rises to converge with the upper band at the band edge, n_2 increases. Also, because the lower band decreases in scattering while the upper band increases in it, μ_2/μ_1 increase. In the Zintl case, convergence by 0.1 eV increases n_2 by about sevenfold. The arrows in Fig. S8 show that, for given increase in n_2 (by sevenfold) and given increase in μ_2/μ_1 (by threefold), it is possible for the overall α to decrease in spite of increased μ_2/μ_1 depending on what the values of μ_2/μ_1 are before and after convergence. If the $\mu_2/\mu_1 = 1$ after convergence, meaning that the two bands are identical, then α increases due to their convergence (arrow 1). For some value that is $\mu_2/\mu_1 > 1$, where the lower band is somewhat lighter than the upper band, band convergence does not change α (arrow 2). But if $\mu_2/\mu_1 >> 1$, meaning that the lower band is far lighter than the upper band, then band convergence can reduce α (arrow 3).

B. More on Carrier Scattering and Transport of the Zintl alloys

Fig. S3 shows that while scattering overall mostly follows the DPS trend of $\tau^{-1} \propto$ DOS, the states within 0.1 eV of the band edge are affected by POS. This is a wide enough width to affect the Seebeck coefficient behavior.

As alloy scattering, grain-boundary scattering, and ionized-impurity scattering were not accounted for in computation, our general overestimation of experimental μ, μ_w, σ, and the PF comes with no surprise. Their absence must be causing some discrepancies in α as well. Comparisons with experimental data are provided in Fig. S4.

Mg_3Sb_2-like Zintl compounds are known to experience significant grain-boundary (GB) scattering that manifests in the form of temperature-activated, hopping-like electronic transport behavior at lower temperatures[5]. The $CaMg_2Sb_2$ samples were indeed highly polycrystalline, which explains the skewed temperature-dependence of mobility in Figs. S4a. Reduction of intrinsic μ by half or more due to GB scattering is routinely reported for alloy thermoelectrics[5–8]. It is well known that electronic transport through GB is a temperature-activated process trending generally as $\mu_{\mathrm{GB}} \propto T^{-0.5}\exp\left(\frac{-\Phi_{\mathrm{GB}}}{k_{\mathrm{B}}T}\right)$ where Φ_{GB} is the potential barrier at the grain boundary[6,8,9]. When we account for this model with parameter adjustment, we see that the experimentally measured mobility behavior of $CaMg_2Sb_2$ is recovered reasonably well. The remaining discrepancy at high temperatures indicates either imperfect model specification for GB scattering or our neglect of ionized-impurity scattering that would arise in experiments due to the Na dopants.

The alloy for which the full band-convergence was achieved, $CaZn_{1.14}Mg_{0.86}Sb_2$, is not only stoichiometrically non-integral but also likely possesses high levels of site disorder. This would introduce disorder scattering that are absent in the perfectly ordered $CaZnMgSb_2$ on which calculations are run. Disorder scattering for electrons is generally modeled as $\mu_{\mathrm{disorder}} \propto T^{-0.5}$[10]. Using this model with suitable parameter adjustment and then applying Matthiessen's rule, we recover the experimental mobility reasonably well as shown in Fig. S4b. At 600 K, disorder roughly halves intrinsic e-ph mobility. The remaining discrepancy at high temperatures again indicates either imperfect model specification for disorder scattering, our neglect of other scattering processes such as ionized-impurity scattering from dopants.

Reduction of intrinsic μ by half or more due to disorder scattering is also routinely reported. In $Mg_2Si_{1-x}Sn_x$ alloys, experimentally fitted effective deformation potential (which lumps the effects of DPS of all kinds: acousiic, optical, intraband and interband[11]) of 13 eV and alloy scattering potential of 0.7 eV corresponded to roughly equal degree of scattering between the two, with one roughly halving the mobility limited by the other[12]. In comparison in the present Zintl alloys, the experimentally inferred scattering potentials were respectively 10 eV and 0.5 eV[13], which indicate similar relative strengths of the two mechanisms.

C. On $Mg_2Si_{1-x}Sn_x$

As mentioned in the main text, $Mg_2Si_{1-x}Sn_x$ is a system whose n-type PF clearly benefits from alloying, peaking around $x \sim 0.7$[12,14,15]. It is presumed that the convergence of its two lowermost conduction bands is responsible for this enhancement, though it occurs at one point (the X-point). We confirm that this convergence occurs by calculating of alloy band structure at $x = 0.75$ using HSE06[16,17] hybrid functional and BandUP[2,3], shown in Fig. S9. However, we cannot positively confirm here whether band convergence indeed is the (primary) reason because the alloys are metallic at the plain DFT level even with $x \sim 0.5$, and hence not amenable to an EPW study. That said, we find a few other reasons that could be primarily responsible for the peak PF around $x \sim 0.7$.

First, the band gap substantially decreases towards the Sn-end ($x = 1$). As computed with the HSE06 hybrid functional[16,17] with SOC, the band gap is 0.53 eV for Mg_2Si, 0.19 eV for $Mg_2Si_{0.25}Sn_{0.25}$ (which is already fairly small) where the bands are close to converging, and 0.02 eV for Mg_2Sn (essentially semimetallic). Therefore, increasing bipolar effect is likely a big reason that the PF falls toward the Sn-end as x increases beyond some optimal value.

Second, the reason that the PF increases with x from the Si-end ($x = 0$) toward $x \sim 0.7$ may at least partly have to do with the fact that, in Mg_2Si, the converging higher-energy band is more favorable for thermoelectrics than the lower-energy band. The latter's profile is approximately $m_\parallel = 0.19$ and $m_\perp = 0.58$ for $\frac{m_\perp}{m_\parallel} = 3.1$, while the former's profile is $m_\parallel = 0.13$ and $m_\perp = 0.97$ for $\frac{m_\perp}{m_\parallel} = 7.5$. The former has a smaller curvature mass in the light direction as well as higher degree of anisotropy, both of which benefit thermoelectricity. As x continues to increase, this band continues to shift downward, which would likely improve the PF perhaps even unto the Sn-end if not for bipolar effect.

Third, as mentioned in the previous section, the experimentally fitted effective deformation potential for $Mg_2Si_{1-x}Sn_x$ was greater than that of $CaZn_{2-x}Mg_xSb_2$, at 13 eV against 10 eV. This means that, unless intraband deformation potential is significantly stronger in $Mg_2Si_{1-x}Sn_x$ than in the Zintl system, it is unlikely that interband scattering is relatively weaker in $Mg_2Si_{1-x}Sn_x$, and therefore possibly just as ineffective for improving thermoelectric performance.

Of course, it remains entirely possible that interband scattering is simply weaker than seen in the Zintl system, only it is challenging to positively confirm. Moreover, the two bands are not as disparate in shapes as the two bands in the Zintl system, which bodes better for thermoelectric performance once they converge in spite of interband scattering. In these cases, band convergence would play a major part in performance improvement in the $Mg_2Si_{1-x}Sn_x$ alloys.

In all, there are likely many different kinds of changes made to the electronic structure of the system when the alloy is created experimentally, clouding whether energy convergence of the bands is the primary let alone the sole reason that the performance peaks at some intermediate x, until it can be directly tested.

IV. SUPPLEMENTARY REFERENCES

[1] Park, J., Xia, Y., Ozoliņš, V., Jain, A. Optimal Band Structure for Thermoelectrics with Realistic Scattering and Bands. *npj Comp. Mater.* **7**, 43 (2021).

[2] Medeiros, P. V. C., Stafström, S., Björk, J. Effects of extrinsic and intrinsic perturbations on the electronic structure of graphene: Retaining an effective primitive cell band structure by band unfolding. *Phys. Rev. B* **89**:041407(R) (2014).

[3] Medeiros, P. V. C., Tsirkin, S. S., Stafström, S., Björk, J. Unfolding spinor wave functions and expectation values of general operators: Introducing the unfolding-density operator. *Phys. Rev. B* **91**:041116(R) (2015).

[4] Nolas, G. S., Sharp, J., Goldsmid, H. J. *Thermoelectrics.* Springer (2001).

[5] Kuo, J. J. et al. Grain boundary dominated charge transport in Mg_3Sb_2-based compounds. *Energy Environ. Sci.* **11**:429–434 (2018).

[6] Slade, T. J. et al. Understanding the thermally activated charge transport in $NaPb_mSbQ_{m+1}$ (Q = S, Se, Te) thermoelectrics: weak dielectric screening leads to grain boundary dominated charge carrier scattering. *Energy Environ. Sci.* **13**:1509–1518 (2020).

[7] Dylla, M. T., Kuo, J. J., Witting, I., Snyder, G. J. Grain Boundary Engineering Nanostructured $SrTiO_3$ for Thermoelectric Applications. *Adv. Mater. Interfaces* **6**:1900222 (2019).

[8] de Boor, J. et al. Microstructural effects on thermoelectric efficiency: A case study on magnesium silicide. *Acta Mater.* **77**:68–75 (2014).

[9] Wang, S. et al. Grain boundary scattering effects on mobilities in p-type polycrystalline SnSe. *J. Mater. Chem. C* **5**:10191–10200 (2017).

[10] Montgomery, D. S. Disorder scattering and electron mobility in $Hg_{1-x}Cd_xTe$. *J. Phys. C: Solid State Phys.* **16**:2923–2934 (1983).

[11] Wang, H., Pei, Y., LaLonde, A. D., Snyder, G. J. *Material Design Considerations Based on Thermoelectric Quality Factor*, pages 3–32. Springer, Berlin, Heidelberg, (2013).

[12] Liu, X. et al. Low Electron Scattering Potentials in High Performance $Mg_2Si_{0.45}Sn_{0.55}$ Based Thermoelectric Solid Solutions with Band Convergence. *Adv. Energy Mater.* **3**:1238–1244 (2013).

[13] Wood, M., Aydemir, U., Ohno, S., Snyder, G. J. Observation of valence band crossing: the thermoelectric properties of $CaZn_2Sb_2$–$CaMg_2Sb_2$ solid solution. *J. Mater. Chem. A* **6**:9437–9444 (2018).

[14] Zaitsev, V. K. et al. Highly effective $Mg_2Si_{1?x}Sn_x$ thermoelectrics. *Phys. Rev. B* **74**:045207 (2006).

[15] Liu, W. et al. Convergence of Conduction Bands as a Means of Enhancing Thermoelectric Performance of n-Type $Mg_2Si_{1-x}Sn_x$ Solid Solutions. *Phys. Rev. Lett.* **108**:166601 (2012).

[16] Heyd, J., Scuseria, G. E., Ernzerhof, M. Hybrid functionals based on a screened Coulomb potential. *J. Chem. Phys.* **118**:8207–8215 (2003).

[17] Heyd, J., Scuseria, G. E., Ernzerhof, M. Efficient hybrid density functional calculations in solids: Assessment of the Heyd–Scuseria–Ernzerhof screened Coulomb hybrid functional. *J. Chem. Phys.* **121**:1187–1192 (2004).
